# Out of Africa: a rare case report of concurrent rupture of the right sinus of Valsalva aneurysm into the interventricular septum and the right atrium

**DOI:** 10.1093/ehjimp/qyae065

**Published:** 2024-07-03

**Authors:** Mauer A A Gonçalves, Humberto Morais, Ana Feijão, Lorete Cardona

**Affiliations:** Luanda Medical Center, Luanda, Angola; Centro de Estudos Avançados em Educação e Formação Médica, Faculdade de Medicina da Universidade Agostinho Neto, Luanda, Angola; Centro de Estudos Avançados em Educação e Formação Médica, Faculdade de Medicina da Universidade Agostinho Neto, Luanda, Angola; Cardiology Department, Hospital Militar Principal Instituto Superior, Luanda, Angola; Luanda Medical Center, Luanda, Angola; Luanda Medical Center, Luanda, Angola

**Keywords:** Sinus of Valsalva aneurysm, echocardiography, computed tomography

A 51-year-old male presented with dyspnoea, fatigue, and lower limb oedema. His history included two years of poorly managed hypertension and a diagnosis of AIDS three months ago, for which he was on antiretroviral therapy. Physical examination revealed a grade III/IV systolic murmur, bibasal rales, and bilateral lower limb oedema. Laboratory results showed NT-Pro-BNP at 6819.1 pg/mL, creatinine at 2.17 mg/dL, and C-reactive protein at 63.6 mg/dL. An ECG indicated complete atrioventricular block (AVB). Transthoracic echocardiography revealed a right sinus of Valsalva aneurysm (RSOVA) dissecting into the interventricular septum (IVS) (*Panel A* asterisk; Supplementary data online, *Movie S1*) and rupturing into the right atrium (RA). (*Panel B*; Supplementary data online, *Movie S2*). Color Doppler studies confirmed turbulent blood flow from the aorta to the aneurysm (*Panel C*; Supplementary data online, *Movies S3* and *S4*) and from the aneurysm to the RA. (*Panel D*; Supplementary data online, *Movie S5*). Computed tomography (CT) imaging corroborated these findings, showing RSOVA (*Panel E*) dissecting into the IVS, *Panel F*) and rupturing into the RA, indicating at least two rupture sites (*Panels G* and *H*). The patient was referred for surgical treatment and placement of a permanent pacemaker, but he died while awaiting surgery.

**Figure:**
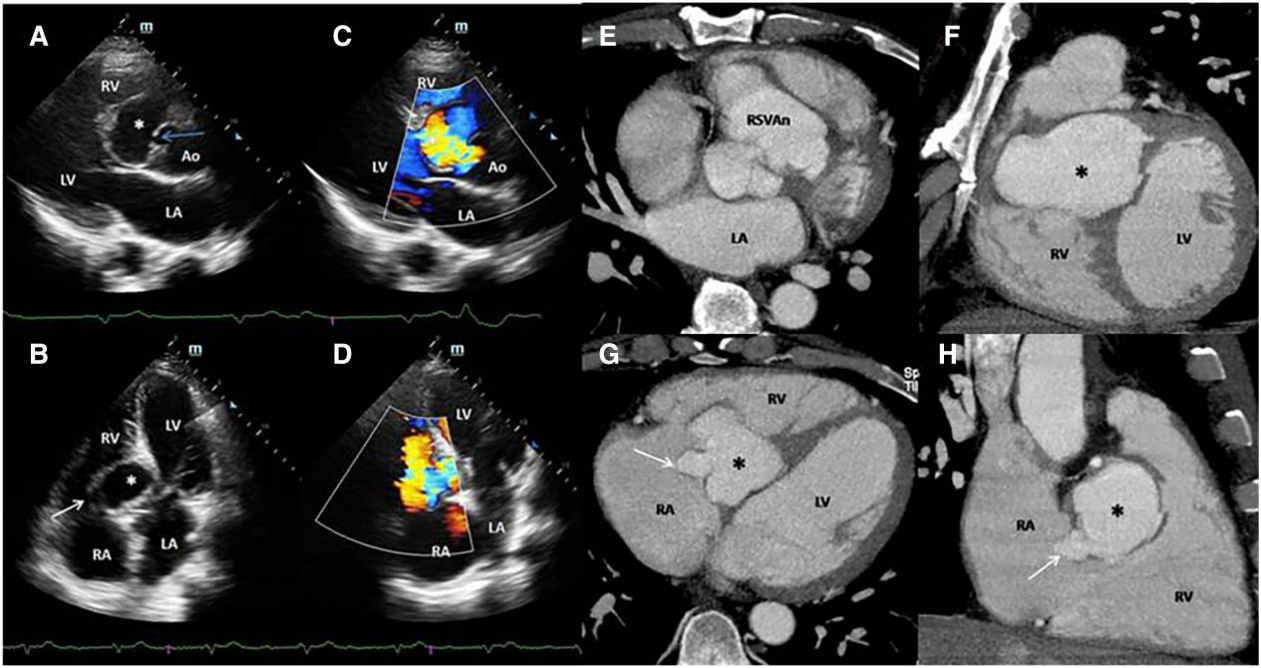


SOVA is a rare but well-known cardiac anomaly. Symptoms typically arise when SOVA ruptures or compresses adjacent structures, leading to heart failure, aortic regurgitation, conduction disorders. In sub-Saharan Africa, rupture into the IVS is the most common complication, often associated with AVB. Echocardiography is the initial imaging modality for suspected SOVA rupture, while CT provides detailed anatomical delineation, especially in cases with multiple ruptures or extensive IVS dissection. Simultaneous rupture of the SOVA into the IVS and left, right or both ventricles is a sporadic heart condition, with 22 cases described in the literature. Concomitant rupture into SIV and RA is even rarer, with only one case described.

## Supplementary Material

qyae065_Supplementary_Data

## Supplementary data

**Consent:** Informed consent was given by the patient.

**Data availability:** The article's data will be shared on reasonable request to the corresponding author.

## Lead author biography

My name is Mauer Gonçalves, I´m specialist in Cardiology. I hold a degree in Cardiopneumology from the Lisbon School of Health Technologies and a degree in Medicine from the Faculty of Medicine at Agostinho Neto University (UAN). MBA in Healthcare Management. PhD in Biomedical Sciences. Faculty member and researcher at the Medical School, UAN. Coordinator of the Advanced Studies Center in Medical Education and Training, Medical School, UAN.

